# Efficacy of fingolimod and interferon beta-1b on cognitive, MRI, and clinical outcomes in relapsing–remitting multiple sclerosis: an 18-month, open-label, rater-blinded, randomised, multicentre study (the GOLDEN study)

**DOI:** 10.1007/s00415-017-8642-5

**Published:** 2017-10-23

**Authors:** Giancarlo Comi, Francesco Patti, Maria Assunta Rocca, Flavia Caterina Mattioli, Maria Pia Amato, Paolo Gallo, Diego Centonze, Carlo Pozzilli, Francesco Saccà, Florian Then Bergh, Marta Bartezaghi, Renato Turrini, Massimo Filippi, For the Golden Study Group, Francesco Patti, Clara Grazia Chisari, Girolama Alessandra Marfia, Diego Centonze, Vincenzo Brescia Morra, Ruggero Capra, Carlo Pozzilli, Valentina Bianchi, Angelo Ghezzi, Marco Roscio, Giancarlo Comi, Francesca Sangalli, Erika Pietrolongo, Ada Francia, Maura Chiara Danni, Ugo Nocentini, Placido Bramanti, Gioacchino Tedeschi, Davide Maimone, Luigi Maria Edoardo Grimaldi, Elio Angelo Scarpini, Antonio Uccelli, Maria Pia Amato, Mariarosa Rottoli, Stefano Ruggieri, Maria Trojano, Roberto Bergamaschi, Florian Then Bergh, Mathias Buttmann, Peter Rieckmann, Ali Safavi

**Affiliations:** 1 Department of Neurology, Institute of Experimental Neurology, San Raffaele Hospital, Milan, Italy; 2 “GF Ingrassia”, Section of Neurosciences, Department of Medical, Surgery Science and Advanced Technology, MS Center, University Hospital, Catania, Italy; 3 Neuroimaging Research Unit, Division of Neuroscience, Institute of Experimental Neurology, San Raffaele Scientific Institute, Vita-Salute San Raffaele University, Milan, Italy; 4 Neuropsychology Unit, Spedali Civili of Brescia, Brescia, Italy; 5 Department NEUROFARBA, University of Florence, Florence, Italy; 6 Department of Neurosciences, Multiple Sclerosis Centre, Veneto Region (CeSMuV), University Hospital of Padova, Padua, Italy; 7 Multiple Sclerosis Research Unit, Department of Systems Medicine, Tor Vergata University, Rome, Italy; 8 Unit of Neurology and Neurorehabilitation, IRCCS Istituto Neurologico Mediterraneo (INM) Neuromed, Pozzilli, Italy; 9 Department of Neurology and Psychiatry, Multiple Sclerosis Centre, S. Andrea Hospital, Sapienza University, Rome, Italy; 10 Department of Neurosciences, Odontostomatological and Reproductive Sciences, University Federico II, Naples, Italy; 11 Department of Neurology, University of Leipzig, Leipzig, Germany; 12 Novartis Farma, Origgio, Varese, Italy

**Keywords:** Fingolimod, Interferon beta-1b, Cognitive impairment, Brief repeatable battery test, Brain atrophy, Delis–Kaplan executive function test

## Abstract

Cognitive impairment (CI) affects 40–65% of multiple sclerosis (MS) patients. This study attempted evaluating the effects of fingolimod and interferon beta-1b (IFN β-1b) on CI progression, magnetic resonance imaging (MRI) and clinical outcomes in relapsing–remitting MS (RRMS) patients over 18 months. The GOLDEN study was a pilot study including RRMS patients with CI randomised (2:1) to fingolimod (0.5 mg daily)/IFN β-1b (250 µg every other day). CI was assessed via Rao’s Brief Repeatable Battery and Delis–Kaplan Executive Function System test. MRI parameters, Expanded Disability Status Scale scores and relapses were measured. Overall, 157 patients were randomised, of whom 30 discontinued the study (fingolimod, 8.49%; IFN β-1b, 41.18%; *p* ≤ 0.0001). Patients randomised to fingolimod had more severe clinical and MRI disease characteristics at baseline compared with IFN β-1b. At Month (M) 18, both treatment groups showed improvements in all cognitive parameters. At M18, relapse rate, total number and volume of T2/T1 gadolinium-enhancing lesions were higher with IFN β-1b, as well as the percentage brain volume change during the study. Safety and tolerability of both treatments were similar to previous studies. Both treatments showed improvements in cognitive parameters. Fingolimod demonstrated significantly better effects on MRI parameters and relapse rate. Imbalance in baseline characteristics and the drop-out pattern may have favoured IFN β-1b. A longer duration trial may be needed to observe the complete expression of differential effects on CI scales reflecting the between-groups differences on MRI. Although limited in size, the GOLDEN study confirms the favourable benefit–risk profile of fingolimod reported in previous studies.

## Introduction

Multiple sclerosis (MS) is a progressive demyelinating disease of the central nervous system (CNS) that results in motor, cognitive and neuropsychiatric impairment [1]. Approximately 40–65% of patients with MS experience symptoms of cognitive impairment (CI), which can affect complex attention, information processing speed, visuospatial memory and executive functions [2, 3]. CI may occur early in the disease course and can lead to considerable deterioration in patients’ quality of life [4]. Currently, there is no proven effective rehabilitation programme or symptomatic treatment for MS-related CI [5, 6].

Disease-modifying therapies (DMTs) approved for MS treatment have proven efficacy in terms of clinical (relapses and disability progression) and magnetic resonance imaging (MRI) (lesion formation and atrophy evolution) parameters [7–9]. However, most of the pivotal, randomised trials on DMTs did not include cognitive endpoints; thus, the evidence on the effect, in particular on the effect size, of these DMTs on CI is inconclusive. Moreover, in the clinical trials that did include cognitive assessment, the assessment was often limited to one or two tests for specific cognitive domains, like the Paced Auditory Serial Addition test (PASAT), and did not comprehensively assess all cognitive domains impacted by MS [5]. A complete assessment by means of a battery of validated tests, such as the Rao’s Brief Repeatable Battery (BRB) [10] or the Brief International Cognitive Assessment for MS (BICAMS) [11], would be important, given the multiplicity of cognitive domains impacted by MS-related CI. Furthermore, evaluation of verbal and non-verbal executive functions, by scoring patients’ performance on a specific scale like the Delis–Kaplan Executive Function System (DKEFS) [12] scale or the Stroop Test [13], is also important for complete assessment of CI.

CI is often accompanied by depression, which may further worsen CI and influence its correct evaluation. Therefore, assessment of depression in these patients using a validated scale like the Montgomery–Asberg Rating Scale (MADRS), a widely known clinician-rated assessment tool, is recommended [14].

Several studies also suggest a correlation between MRI measures (white matter and grey matter lesion number and/or volume, global brain and grey matter volume) and CI, which is yet to be fully assessed in randomised, therapeutic trials [2, 15–17].

Once-daily oral fingolimod (Gilenya^®^, Novartis Pharma AG) is a sphingosine-1-phosphate (S1P) receptor modulator approved for the treatment of relapsing forms of MS [7, 18]. Fingolimod acts by reducing the number of recirculating autoreactive T-cells entering the CNS and destroying the myelin sheath, via reducing egress of these lymphocytes from the lymph nodes. Unlike interferons (IFNs), which have an immunomodulatory effect but lack any direct effects on CNS cells, fingolimod crosses the blood–brain barrier and acts directly on the S1P receptors located on these cells, leading to reduction of reactive activation of glia (which may favour naturally occurring remyelination) [19]. This mechanism of action might be responsible for the effects of fingolimod on slowing brain atrophy observed in previous studies (which in turn is possibly associated with CI) [7, 18]. In phase III pivotal studies, fingolimod-treated MS patients developed less brain atrophy versus patients receiving placebo both at Year 1 (−0.50 vs. −0.65%) and at Year 2 (−0.84 vs. −1.31%) in the FREEDOMS study [18], and versus patients receiving interferon beta-1a (IFN β-1a) over 1 year (−0.31 vs. −0.45%) in the TRANSFORMS study [7].

The effect of fingolimod on CI in patients with MS has been assessed using the PASAT in two pivotal phase III, randomised studies—FREEDOMS and TRANSFORMS. In both these studies, a trend towards greater proportion of correct responses on the PASAT-3 was observed in patients treated with fingolimod compared with those receiving placebo (FREEDOMS) or IFN β-1a (TRANSFORMS, where the difference versus IFN β-1a was significant with *p* = 0.049) [20]. An observational, single-centre, open-label, 1-year prospective study conducted by Barak et al. suggested that fingolimod confers cognitive stability in patients with active relapsing–remitting MS (RRMS) [21]. Fingolimod has also shown positive effects on cognitive parameters in various clinical trials and real-world studies [22–24]. However, these effects still need to be assessed in a comprehensive way with respect to all the cognitive domains that are altered during the course of MS as well as in comparison to the effects of standard-of-care DMTs.

The 18-month ‘GOLDEN’ (Fin*gol*imo*d* on cognitiv*e* symptoms and brai*n* atrophy) study aimed at evaluating the effects of treatment with fingolimod and IFN β-1b on CI progression using the BRB and DKEFS scale as well as on MRI and clinical outcomes in patients with RRMS. This side-by-side evaluation was designed to provide pilot evidence for the effect of fingolimod and IFN β-1b on cognitive, MRI and clinical outcomes. A direct comparison between fingolimod and IFN β-1b was not a prespecified objective of this study.

## Methods

### Study design and patients

The GOLDEN study (ClinicalTrials.gov identifier NCT01333501) was an 18-month multicentre, open-label, rater-blinded, randomised, parallel-group pilot study conducted in patients with RRMS. Eligible patients were aged 18–60 years and diagnosed with RRMS (per the 2005 revised McDonald criteria [25]) with active disease and CI at screening. Active disease was defined as at least one clinical relapse in the past year, or two clinical relapses in the past 2 years if there were signs of disease activity in one brain MRI scan performed in the past 6 months. CI was defined as ≥ 1 test of the Rao’s BRB with scores below the tenth percentile of age- and gender-based normative data. Key exclusion criteria included unsatisfactory response with multi-weekly IFNs (IFN β-1a/b), hyperactive forms of MS, Expanded Disability Status Scale (EDSS) score > 5.0, acute MS relapse < 30 days before screening, prior or current diagnosis of major depression according to the Diagnostic and Statistical Manual of Mental Disorders—Text Revision (DSM-IV-TR) and history of any chronic disease of the immune system other than MS.

**Fig. 1:**
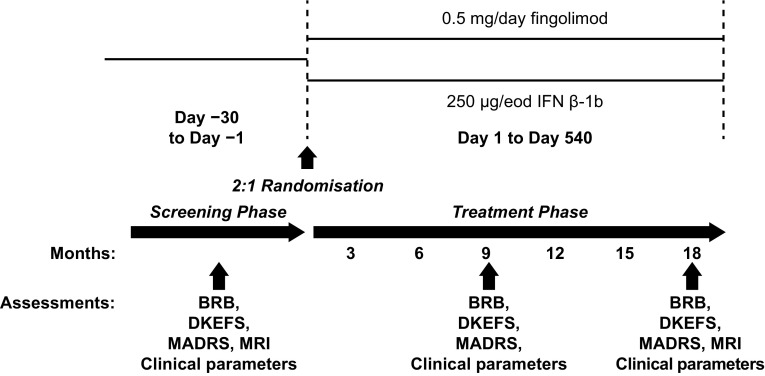
Study design. *BRB* Rao’s brief repeatable battery, *DKEFS* Delis–Kaplan executive function system, *eod* every other day, *MADRS* Montgomery–Asberg Depression Rating Scale, *MRI* magnetic resonance imaging

All patients provided written informed consent before enrolment. The study was conducted in accordance with the International Conference on Harmonisation Guidelines for Good Clinical Practice and the Declaration of Helsinki [26, 27]. The study was conducted at 36 study centres, 28 in Italy (22 recruiting) and 8 in Germany (4 recruiting), and the protocol was approved by the Independent Ethics Committee or Institutional Review Board at each centre. The study included a 1-month screening phase to determine eligibility. At baseline, eligible patients were randomised (2:1) to receive oral fingolimod (0.5 mg/day) or subcutaneous IFN β-1b (250 µg every other day; Fig. 1). Study visits for patient clinical assessment were scheduled at screening, baseline and Months 3, 6, 9, 12, 15 and 18 of treatment.

### Efficacy assessments

CI was assessed using the BRB of neuropsychological tests in MS [28], comprising five tests: Selective Reminding Test [SRT; includes long-term storage (SRT-LTS), consistent long-term retrieval (SRT-CLTR) and delayed recall (SRT-d)]; 10/36 Spatial Recall Test (10/36 SPART) [total correct responses (10/36 SPART-T) and delayed recall (10/36 SPART-DR)]; Symbol Digit Modalities Test (SDMT); PASAT and Word List Generation (WLG) test performed at screening, Month 9 and Month 18. All BRB tests have been validated in Italian and German and are available in two alternate forms (A and B), which were administered according to the scheme A–B–A to reduce the practice effect. Goretti et al. have suggested that, similar to other languages, the Italian B version of the BRB test may be easier than version A but also suggested that the application of the normative values provided in their study can overcome this issue [29]. We therefore considered that the BRB versions A and B are comparable only when the data are normalised. Moreover, normalisation of BRB in German is available only for version A.

Executive functions were assessed using the DKEFS-Sorting test [30], one of the nine tests presented in the DKEFS manual, at screening, Month 9 and Month 18. The DKEFS test consisted of two testing procedures: free sorting and sort recognition. In free sorting, six scores were obtained: confirmed correct sorts for card sets 1 and 2 (or 3 and 4 for version B), sum of confirmed correct sorts, free sorting description score for card sets 1 and 2 (or 3 and 4 for version B) and sum of free sorting description scores. In sort recognition, a description score for card sets 1 and 2 (or 3 and 4 for version B) as well as the sum of description scores of both sets were obtained.

The MADRS [14] was used to assess depression at screening, Month 9 and Month 18.

MRI was performed locally at the participating centres on 1.5 T (or higher) scanners according to a prespecified protocol provided by the central reading facility (Neuroimaging Research Unit, Institute of Experimental Neurology, San Raffaele Scientific Institute, Milan, Italy). MRI parameters [T2-hyperintense, T1-hypointense and T1-enhancing lesions, normalised brain volume (NBV) and percentage brain volume change (PBVC) versus screening scan] were assessed (using central reading) at screening and Month 18. The identification of white matter lesions was performed by consensus of two experienced observers, and the volume of the identified lesions was measured using a semiautomated segmentation technique based on local thresholding (Jim 6.0; Xinapse System, UK). NBV at screening and PBVC during the follow up were measured on precontrast 3D T1-weighted images, using the SIENAx and SIENA software.

Patients were assessed for MS relapses during the course of the study.

EDSS scores were assessed by local raters, specifically trained to minimise variability, at screening, Month 9 and Month 18.

### Safety assessments

Safety assessments included reporting of adverse events (AEs), serious AEs (SAEs), vital signs, physical/neurological examinations, skin examination, laboratory examinations, electrocardiogram (ECG) monitoring (as required) and ophthalmologic examinations.

AEs, SAEs and vital signs were assessed at each study visit. Physical examinations were performed at screening and Months 6, 12 and 18; ophthalmologic examinations were performed at screening and Months 3, 6 and 18; and skin examinations were performed at screening and Month 18.

### Statistical analysis

For the efficacy data analysis, the full analysis set (FAS) population was considered instead of the per-protocol population because of the high drop-out rate, particularly in the IFN β-1b group. The FAS population included all randomised patients who received at least one dose of the study drug and had at least one post-baseline assessment of both primary efficacy variables (i.e., non-missing information on BRB and DKEFS-Sorting test) without any major protocol deviations. The safety population included all randomised patients who received at least one dose of the study drug.

Continuous data were summarised by mean, standard deviation (SD), median, interquartile range, minimum and maximum, and 95% confidence limits (CLs), where applicable. Categorical data were presented by absolute and relative frequencies (*n* and %) or contingency tables. Homogeneity tests were run to assess differences between groups at screening/baseline. The normality of continuous variables was evaluated by means of a Shapiro–Wilk test to perform the *t* test (in case of normal data distribution) or the Wilcoxon–Mann–Whitney test (in case of non-normal data distribution).

Chi square tests were performed for categorical variables, or Fisher’s exact test for cell frequencies < 5.

Differences between groups in each of the BRB tests were analysed by means of analysis of covariance (ANCOVA) on raw scores (no a priori data normalisation of raw scores based on age/gender and education), with propensity score as an independent variable. The following variables were considered in the propensity score adjustment: EDSS score at screening, disease duration, naïve or treated patients’ status, MADRS total score at screening, T2 lesion volume (LV), T1 hypointense LV and number of altered BRB tests at screening.

An ANCOVA model was applied to assess the differences between groups in MRI variables, except for the number of T1-enhancing lesions and T2-hyperintense lesions, considering propensity score as an explanatory variable and screening value as the covariate. The propensity score analysis considered the following factors: EDSS score, disease duration, naïve or treated patients, depression (MADRS score) and number of altered cognitive tests. Differences between groups in number of T1 gadolinium-enhancing (Gd+) lesions and new T2 lesions were assessed by means of a negative binomial regression model, including the same propensity score variables applied for the ANCOVA model on the other MRI variable.

Differences between groups in MADRS scores overtime were analysed by means of a mixed model for repeated measures (MMRM). The explanatory variables in the longitudinal model included treatment, visit, treatment-by-visit interaction, missing data pattern (completers, missing at Month 9, missing at Month 18), propensity score and corresponding baseline. The propensity score analysis considered the following factors: EDSS score, disease duration, naïve or treated patients, number of altered tests, T2 LV and T1 hypointense LV.

The correlation between each BRB test and each MRI variable was performed using Pearson correlation in case of normal distribution of both the considered variables, or Spearman correlation otherwise.

## Results

### Patient disposition, demographic, and baseline characteristics

**Fig. 2:**
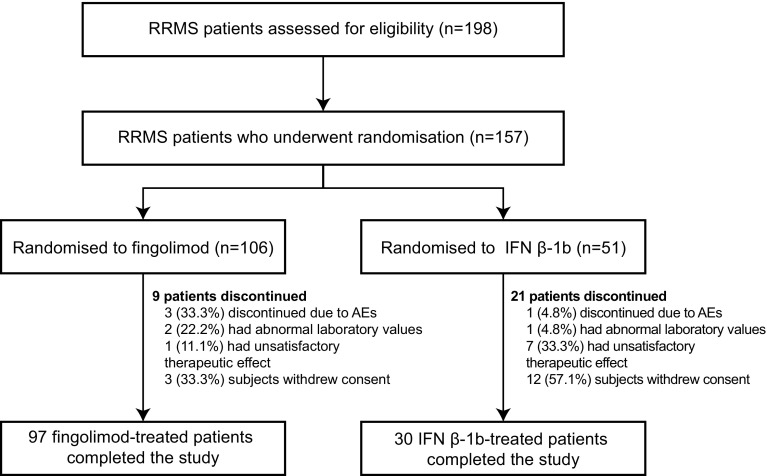
Patient disposition. *AE* adverse event, *IFN β-1b* interferon beta-1b, *RRMS* relapsing–remitting multiple sclerosis

In total, 198 patients were enrolled and screened in the GOLDEN study, of whom 157 were randomised (2:1) to receive either fingolimod (*n* = 106) or IFN β-1b (*n* = 51). Overall, 30 of the randomised patients discontinued the study: 9 (8.49%) patients from the fingolimod group and 21 (41.18%) patients from the IFN β-1b group (*p* ≤ 0.0001) (Fig. 2).

The safety population comprised 151 patients (fingolimod, 104; IFN β-1b, 47), whereas the FAS population comprised 108 patients (fingolimod, 80; IFN β-1b, 28). Approximately 50% of the patients in the FAS were treatment naïve (fingolimod, 47.5%; IFN β-1b, 53.57%; Table 1). The most recent previous treatments included glatiramer acetate, IFN β-1a, IFN β-1b, natalizumab, azathioprine and mitoxantrone.

**Table 1:** Baseline characteristics of patients included in the FAS

	Fingolimod (*n* = 80)	IFN β-1b (*n* = 28)	*p* value
Patient demographics			
Age at screening, years	40.23 (9.09)	37.64 (9.29)	0.2195^a^
Female, *n* (%)	57 (71.25)	19 (67.86)	0.7351^b^
Caucasian^e^, *n* (%)	79 (98.75%)	18 (100.00%)	–
Disease characteristics			
Age at MS diagnosis, years	35.2 (9.22)	32.89 (8.10)	0.2572^a^
Disease duration, years	4.97 (6.67)	4.71 (6.47)	0.5032^a^
Number of relapses in the past year	1.45 (0.79)	1.18 (0.48)	0.1193^a^
Number of relapses in the past 2 years*	1.90 (0.84)	1.54 (0.84)	0.0191^a^
EDSS score at screening*	2.78 (1.34)	2.09 (1.05)	0.0202^a^
Treatment-naïve patients, *n* (%)	38 (47.50)	15 (53.57)	0.5802^b^
MRI variables			
Normalised brain volume, mL*	1391 (94)	1433 (99)	0.0452^c^
Volume of total T2 lesions (mm^3^)	10813.00 (10425.76)	7509.21 (7045.94)	0.1770^a^
Volume of total T1 hypointense lesions (mm^3^)	4076.60 (5317.53)	2090.54 (2217.19)	0.1419^a^
Volume of total T1 enhancing lesions (mm^3^)	63.95 (158.33)	76.89 (146.32)	0.6252^a^
Number of total T1 enhancing lesions	0.75 (1.15)	0.89 (1.91)	0.6260^d^
Cognitive parameters (raw scores)			
SRT-LTS	32.35 (14.36)	37.04 (15.31)	0.1469^c^
SRT-CLTR	22.54 (14.06)	29.36 (17.30)	0.0616^a^
SPART	16.50 (4.78)	17.61 (5.23)	0.1629^a^
SDMT*	40.89 (14.30)	47.39 (16.14)	0.0183^a^
PASAT-3*	30.15 (13.03)	37.82 (14.49)	0.0105^c^
PASAT-2	23.31 (12.59)	27.96 (12.76)	0.0515^a^
SRT-d	6.48 (2.86)	7.18 (2.89)	0.2670^c^
SPART-d	5.80 (2.23)	6.29 (2.34)	0.3354^a^
WLG	19.68 (5.63)	21.25 (6.77)	0.2639^a^
MADRS	10.77 (7.34)	7.93 (6.24)	0.0806^a^

Data represent mean (SD) unless specified otherwise

*EDSS* expanded disability status scale, *FAS* full analysis set, *IFN β-1b* interferon beta-1b, *MADRS* Montgomery–Asberg rating scale, *MRI* magnetic resonance imaging, *MS* multiple sclerosis, *PASAT* paced auditory serial addition test, *SD* standard deviation, *SDMT* symbol digit modalities test, *SPART* 10/36 spatial recall test, *SPART-d* 10/36 spatial recall test-delayed recall, *SRT-LTS* selective reminding test-long-term storage, *SRT-CLTR* selective reminding test-consistent long-term retrieval, *SRT-d* selective reminding test-delayed recall, *WLG* word list generation

*Number of relapses in the past 2 years, EDSS score at baseline and cognitive test scores (PASAT and SDMT) were statistically significant, indicating that IFN β-1b group patients had more severe disease than fingolimod group patients

^a^Wilcoxon two-sample test

^b^Chi-square

^c^ *t* test

^d^Negative binomial regression model

^e^For safety population

Contrary to the safety population where baseline characteristics were similar across the two treatment groups, in the FAS population, patients randomised to receive fingolimod had higher number of relapses in the past 2 years (*p* = 0.0191), higher EDSS scores (*p* = 0.0202), lower NBV (*p* = 0.0452) and worse cognitive test scores [PASAT-3 (*p* = 0.0105) and SDMT (*p* = 0.0183)] compared with the IFN β-1b group (Table 1).

**Fig. 3:**
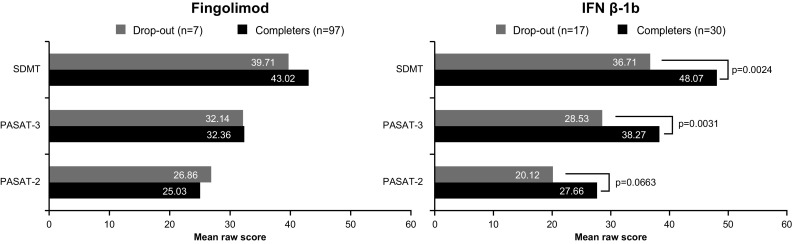
SDMT and PASAT scores at screening for patients who completed the treatment versus those who dropped out of the study (safety population). *IFN β-1b* interferon beta-1b, *PASAT* paced auditory serial addition test, *SDMT* symbol digit modalities test. The figure represents mean values of PASAT and SDMT raw scores. The *p* value was calculated using the *t* test for SDMT and PASAT-2 in IFN group and PASAT-3 in fingolimod group; otherwise, the Wilcoxon two-sample test was used

There were statistically significant differences between the baseline PASAT and SDMT (and WLG) scores of patients who completed the study versus those who dropped out of the study in the IFN β-1b group: in this group, patients who later dropped out had more severe baseline scores than the completers, whereas this effect was not observed for the patients in the fingolimod group (only a non-statistically significant trend was noted for SRT; Fig. 3).

The total duration of treatment exposure was 537.00 ± 105.77 (range 4–650) days for fingolimod and 420.38 ± 183.37 (range 1–568) days for IFN β-1b (*p* ≤ 0.0001).

### Efficacy results

#### Cognitive function

**Fig. 4:**
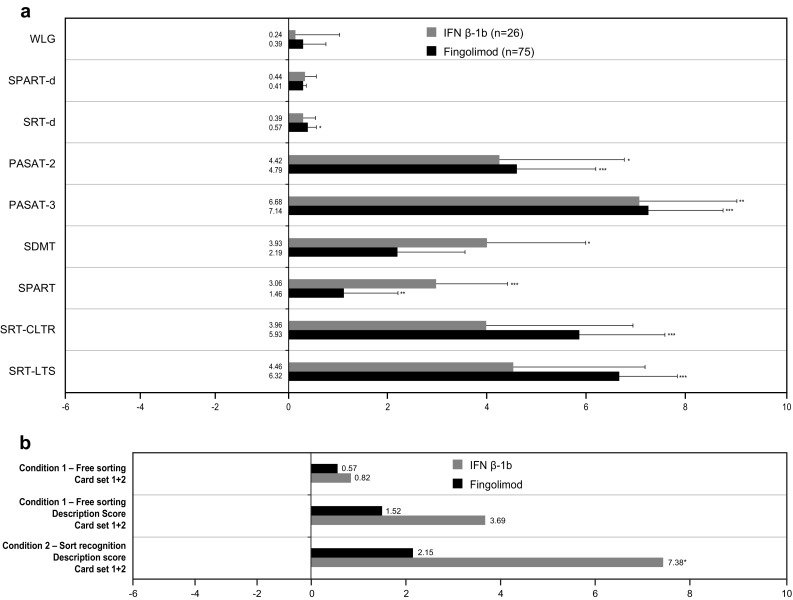
Cognitive impairment test results **a** Rao’s BRB test: ANCOVA LS mean changes Month 18 versus screening (FAS population) *p < 0.05; **p < 0.01; ***p < 0.001. *ANCOVA* analysis of covariance, *BRB* brief repeatable battery, *FAS* full analysis set, *IFN β-1b* interferon beta-1b, *LS* least squares, *M* month, *PASAT* paced auditory serial addition test, *SDMT* symbol digit modalities test, *SE* standard error, *SPART* 10/36 spatial recall test, *SPART-d* 10/36 spatial recall test-delayed recall, *SRT-LTS* selective reminding test-long-term storage, *SRT-CLTR* selective reminding test-consistent long-term retrieval, *SRT-d* selective reminding test-delayed recall, *WLG* Word List Generation, **b** DKEFS-sorting test: ANCOVA model estimated mean (LSmean) changes at Month 18 versus screening (FAS population). *p < 0.05; **p < 0.01. Note: Condition 1—free sorting: fingolimod 66, IFN β-1b 27. Condition 1—free sorting description: fingolimod 65, IFN β-1b 27. Condition 2–sort recognition: fingolimod 50, IFN β-1b 21. *ANCOVA* analysis of covariance, *DKEFS* Delis–Kaplan executive function test, *FAS* full analysis set, *IFN β-1b* interferon beta-1b, *LS* least squares, *M* month

For the BRB test, both treatment groups showed improvements in mean changes of all parameters from screening to Month 18: SRT-d (*p* = 0.0163 for fingolimod; *p* = 0.3270 for IFN β-1b), PASAT-2 (*p* = 0.0002 for fingolimod; *p* = 0.0413 for IFN β-1b), PASAT-3 (*p* < 0.0001 for fingolimod; *p* = 0.0022 for IFN β-1b), SDMT (*p* = 0.0540 for fingolimod; *p* = 0.0445 for IFN β-1b), SPART (*p* = 0.0058 for fingolimod; *p* = 0.0009 for IFN β-1b), SRT-CLTR (*p* = 0.0001 for fingolimod; *p* = 0.1246 for IFN β-1b), SRT-LTS (*p* < 0.0001 for fingolimod; *p* = 0.0534 for IFN β-1b), SPART-d (*p* = 0.0502 for fingolimod; *p* = 0.2210 for IFN β-1b) and WLG (*p* = 0.5017 for fingolimod; *p* = 0.8128 for IFN β-1b) (Fig. 4a). No significant differences were detected between the treatment groups in the mean changes in all parameters from screening to Month 18.

#### Executive function

Similar to the BRB test, both treatment groups showed improvements in the mean changes from screening to Month 18 for the components of the DKEFS Sorting test (Fig. 4b). No significant differences were detected between the treatment groups in the mean changes from screening to Month 18 for components of DKEFS-Sorting test.

#### Depression

The MADRS total score was higher (though not significantly) in the fingolimod group at screening (10.77 ± 7.34 vs. 7.93 ± 6.24; *p* = 0.0806). At Month 18, fingolimod-treated patients still exhibited higher scores compared with IFN β-1b-treated patients; however, changes versus screening indicated slight improvement only in the fingolimod group [−0.68 ± 7.57, 95% CL (− 2.45, 1.08) versus a change of 0.30 ± 5.63, 95% CL (− 1.93, 2.52) in the IFN β-1b group], although the difference was not statistically significant (*p* = 0.3291).

#### MRI results

**Fig. 5:**
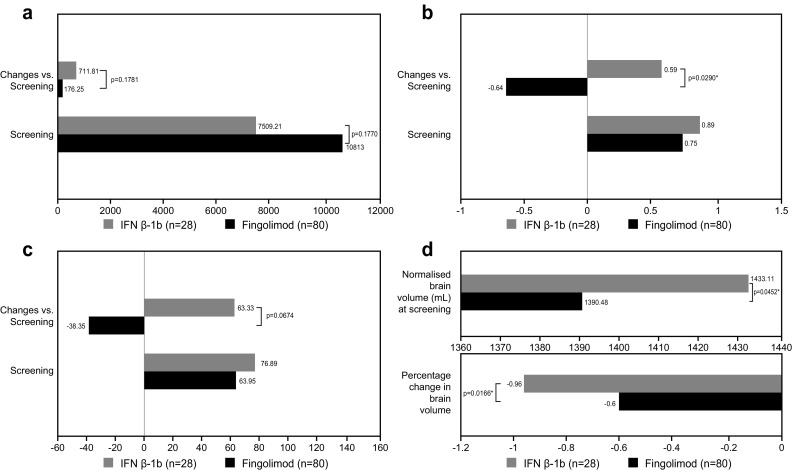
MRI measures. **a** Total T2 lesion volume (mm^3^), **b** number of T1 Gd+ lesions, **c** volume of T1 Gd+ lesions (mm^3^), and **d** brain volume (mL). *p* values for the changes were calculated using an ANCOVA model, except for the number of T1 Gd+ lesions, which was computed by using a negative binomial regression model. At baseline, the *p* values were calculated using the Wilcoxon two-sample test, except for the brain volume, which was calculated using the *t* test. *p < 0.05 within group. *ANCOVA* analysis of covariance, *Gd+* gadolinium enhancing, *IFN β-1b* interferon beta-1b, *M* month, *MRI* magnetic resonance imaging

T2 LV at screening was higher in the fingolimod group versus the IFN β-1b group (*p* = 0.1770); although the mean T2 LV decreased in both groups from screening to Month 18, when taking the deltas into account, i.e. only patients who had both values, on average it increased from screening to Month 18 in both treatment groups (Fig. 5a). At Month 18, patients in the IFN β-1b group presented with more new T2 lesions (3.33 ± 4.44 vs. 1.25 ± 2.05) than those in the fingolimod group (*p* = 0.0276 between groups).

The number and volume of Gd+ lesions at screening were similar in the two groups; both number and volume decreased in patients treated with fingolimod (significantly for the number of lesions, *p* = 0.0316) and increased in patients treated with IFN β-1b. The between-group difference was significant for the number of T1 Gd+ lesions (*p* = 0.0290; Fig. 5b, c).

NBV at screening was significantly lower in the fingolimod group versus the IFN β-1b group (1391 vs. 1433 mL; *p* = 0.0452). During the study, the PBVC from screening to Month 18 in the IFN β-1b group (−0.96% ± 0.71%) was larger than that in the fingolimod group (−0.60% ± 0.83%; Fig. 5d), and the between-group difference was statistically significant in favour of fingolimod (*p* = 0.0166).

No significant correlation was found between the effects of treatment on MRI parameters and on the various tests of the BRB or DKEFS.

#### Relapses and EDSS

**Fig. 6:**
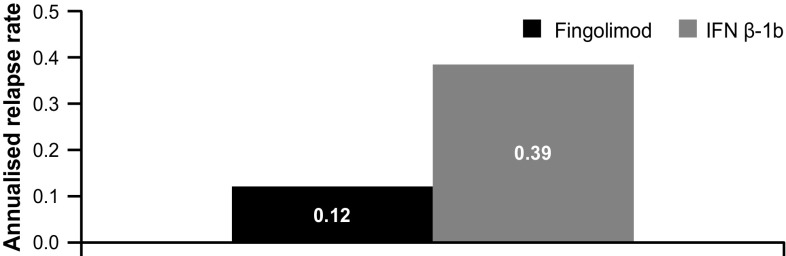
MS relapse rate (safety population). *IFN β-1b* interferon beta-1b, *MS* multiple sclerosis

The proportion of patients with at least one relapse during the study period was significantly higher in the IFN β-1b group than in the fingolimod group (31.91 vs. 15.38%, *p* = 0.0199; Table 2). Moreover, the annualised relapse rate was also higher in the IFN β-1b group than in the fingolimod group (0.39 vs. 0.12; Fig. 6).

**Table 2:** MS relapse (safety population)

	Fingolimod (*n* = 104)	IFN β-1b (*n* = 47)	*p* value*
No. of patients with at least one relapse	16 (15.38%)	15 (31.91%)	0.0199
Of above, patients with			
1 relapse	13 (81.25%)	10 (66.67%)	
2 relapses	2 (12.50%)	3 (20.00%)	
3 relapses	1 (6.25%)	2 (13.33%)	

*Chi-square test

EDSS scores remained virtually stable over the 18-month study period, with very small changes in scores at Month 18 versus screening, of 0.12 ± 0.84 [95% CL (− 0.07, 0.31)] in the fingolimod group and 0.19 ± 0.54 [95% CL (− 0.03, 0.40)] in the IFN β-1b group.

### Safety results

Overall, AEs were reported in 79.81% of patients treated with fingolimod and 59.57% treated with IFN β-1b (Table 3a). No deaths were reported during the study.

**Table 3:** AEs and SAEs in the safety set

*n* (%)	Fingolimod (*n* = 104) (%)	IFN β-1b (*n* = 47) (%)	*p* value*
(a) Summary of AEs (safety population)			
Number of patients with at least one AE	83 (79.81)	28 (59.57)	0.0091
Number of patients with at least one SAE	9 (8.65)	1 (2.13)	0.1354
Number of patients with at least one AE suspected to be study drug related	37 (35.58)	10 (21.28)	0.0789
Number of patients with at least one AE leading to discontinuation	5 (4.81)	3 (6.38)	0.6891

SOC ≥ 5%, *n* (%)	Fingolimod (*n* = 104) (%)	IFN β-1b (*n* = 47) (%)	
(b) AEs by SOC ≥ 5% (safety population)			
Number of patients with at least one AE	83 (79.81)	28 (59.57)	
Blood and lymphatic system disorders	7 (6.73)	0	
Eye disorders	8 (7.69)	1 (2.13)	
Gastrointestinal disorders	22 (21.15)	5 (10.64)	
General disorders and administration site conditions	17 (16.35)	10 (21.28)	
Infections and infestations	29 (27.88)	9 (19.15)	
Injury, poisoning and procedural complications	6 (5.77)	3 (6.38)	
Investigations	26 (25.00)	9 (19.15)	
Metabolism and nutrition disorders	8 (7.69)	2 (4.26)	
Musculoskeletal and connective tissue disorders	11 (10.58)	5 (10.64)	
Nervous system disorders	19 (18.27)	12 (25.53)	
Psychiatric disorders	13 (12.50)	6 (12.77)	
Renal and urinary disorders	6 (5.77)	4 (8.51)	
Respiratory, thoracic and mediastinal disorders	6 (5.77)	3 (6.38)	
Skin and subcutaneous tissue disorders	11 (10.58)	0	
Vascular disorders	6 (5.77)	1 (2.13)	

*AE* adverse event, *IFN β-1b* interferon beta-1b, *SAE* serious adverse event

*Chi-square test

The proportion of patients with SAEs was higher in the fingolimod group versus the IFN β-1b group (8.65 vs. 2.13%), with one SAE suspected of being related to the study treatment in the fingolimod group (second-degree atrioventricular block after first dose of the drug, in a patient who underwent overnight hospitalisation and then continued treatment with fingolimod without problems), while the proportion of patients discontinuing the study due to AEs was higher in the IFN β-1b group (6.38 vs. 4.81%). The most commonly reported (first five) system organ classes (SOCs) in both groups were ‘infections and infestations’ (primarily nasopharyngitis and influenza), ‘investigations’ (primarily alanine aminotransferase [ALT], blood cholesterol and transaminase increases for fingolimod; transaminase, blood triglyceride and cholesterol increases for IFN β-1b), ‘nervous system disorders’ (mostly headache for fingolimod and MS relapse for IFN β-1b), ‘gastrointestinal disorders’(only one case of diarrhoea was thought to be related to treatment, in the fingolimod group), ‘general disorders and administration site conditions’ (fever, fatigue and influenza-like illnesses more frequent in the IFN β-1b group). Of the 11 cases of the SOC ‘Skin and subcutaneous tissue disorders’ that have been reported only among patients treated with fingolimod, two were considered possibly related to the treatment: one case of psoriasis and one case of alopecia (Table 3b).

## Discussion

Cognitive dysfunction is a common clinical problem in MS and is associated with functional impairment leading to deterioration in patients’ quality of life [4]. Thus, a number of studies have been performed on cognitive dysfunction in patients with MS; however, most of the evidence supporting the effect of DMTs on cognitive dysfunction comes from observational, often uncontrolled/non-randomised, studies using single tests that typically assess only one cognitive domain [5, 31–33]. Moreover, controlled trials investigating the effect of DMTs in MS were primarily designed to evaluate clinical outcomes, and cognitive dysfunction was assessed as a secondary outcome or only in a subgroup of patients [5].

This is the first randomised, double-blind study to prospectively and comprehensively assess CI in MS patients treated with fingolimod versus an active control (IFN β-1b). A number of characteristics made our study design quite robust in assessing CI in MS patients over the given period of time. First, as described earlier, we have used the full BRB test, which is a sensitive and widely used tool in clinical practice, and also assesses executive functions with the DKEFS scale. Second, patients were included in the study only if they already presented CI at screening. This ‘enrichment design’ is important, since it is known that preserved cognitive function can remain stable over a long time in MS, whereas incipient cognitive decline seems to be widespread and progressive in nature [34]. Thus, trials in cognitively unselected populations have lower chances to detect the differences in CI that will accumulate in such a population over the course of the study, particularly if the study duration is short and does not run over many years, whereas these chances were maximised with our enriched population design. Third, in order to prevent the learning of specific test stimuli and thus potentially mitigate practice effects, all the tests of the BRB were provided at screening, Month 9 and Month 18 in two alternate forms (A and B, scheme of administration: A–B–A). Fourth, a standard MRI acquisition protocol was followed across all the sites to ensure uniform quality of the scans across the sites; scans were then analysed at a central MRI evaluation centre by physicians who were unaware of the study-group assignments.

This notwithstanding, the following factors were taken into account when interpreting the results of our study, particularly with regard to the less objective endpoints like cognitive scales, compared to the more objective endpoints like MRI. In addition, one has to remember that the study was neither powered nor designed to serve as a direct comparison between fingolimod and IFN β-1b on cognitive scales, but rather to provide side-by-side, pilot evidence. First of all (and contrary to the safety population, where there were no differences between the treatment groups, which indicates that randomisation did work), in the FAS population patients in the fingolimod group had more severe disease characteristics at baseline compared with patients in the IFN β-1b group, significantly so in terms of number of relapses in the past 2 years, EDSS score, NBV and cognitive test scores (PASAT and SDMT). Second, as expected, a considerably higher percentage of patients treated with IFN β-1b versus fingolimod prematurely discontinued treatment (41.18 vs. 8.49%), with the main reasons for discontinuation being ‘unsatisfactory therapeutic effect’ and ‘withdrawal of consent’. One implication is that patients in the IFN β-1b group with more severe disease abandoned the trial, as confirmed by the difference between the randomised population and the FAS population. A second implication is that patients in the IFN β-1b group who completed the study and had Month 18 cognitive scores available were most probably those who were responding better to that treatment compared to the fingolimod group. This is supported by the statistically significant differences between the baseline PASAT and SDMT (and WLG) scores of patients who completed the study versus those who dropped out of the study in the IFN β-1b group: in this group patients who later dropped out had more severe baseline scores than the completers, whereas this effect was not observed for the patients in the fingolimod group (only a non-statistically significant trend was noted for SRT; Fig. 3).

Our results show that both fingolimod and IFN β-1b improved all cognitive domains affected by MS, as evaluated through the various tests of the BRB (SRT-d, SRT-LTS, SRT-CLTR, SPART, SPART-d, SDMT, PASAT and WLG) with some differences in the improvement pattern. Fingolimod showed the best effect on PASAT and SRT scores, whereas IFN β-1b showed the best effect on SDMT scores. In this regard, it is worth noting that PASAT is a difficult and demanding test of the battery, relatively more difficult than the SDMT, and is sensitive even to early cognitive changes in patients where room for improvement is still limited [35, 36]. A longitudinal correlational research study suggested that PASAT is particularly sensitive to inflammatory activity measured by Gd+ enhancement in otherwise physically stable patients with MS [37]. Hence, the observed effect of fingolimod on PASAT in this study is consistent with the effect observed on Gd+ lesions.

In general, our results are supported by previous studies evaluating the effect of IFN or fingolimod on CI in real-world setting using a non-randomised design. A 3-year, open-label, prospective, observational study [COGnitive Impairment in Multiple Sclerosis (COGIMUS)] showed that the proportion of cognitively impaired patients treated with IFN β-1a remained stable over the 3 years of treatment in at least three tests of the BRB and the Stroop Color-Word Task [33]. In another 1-year, open-label study, treatment with IFN β-1b in RRMS patients led to improved performance in the complex attention, concentration and visual learning and recall domains compared with patients with RRMS matched for neurological disability [31]. In a post hoc analysis of pooled data from a fingolimod phase III trial in patients with RRMS, fingolimod treatment resulted in early and sustained improvement in cognition, as measured by the change in PASAT-3 scores over 6, 12 and 24 months [23]. Results of a multicentre, examiner-blinded, prospective trial also showed significant improvement in cognitive function from the sixth month of initiation of fingolimod in patients with RRMS [24]. Additionally, real-world studies in patients with RRMS have shown a positive impact of fingolimod treatment on cognitive parameters [22]. In a study comparing effectiveness of natalizumab and fingolimod treatment on cognitive functions by using the BRB tests in patients with RRMS from clinical practice, fingolimod was found to be more effective than natalizumab in improving cognitive function [38]. However, our study provides novel information on the topic. Namely, we show that there is not necessarily any direct correlation between the effects of a given treatment on MRI parameters (particularly on brain atrophy, which in turn has been shown to correlate with a beneficial effect on CI) and on the multiplicity of tests that compose the BRB or DKEFS. In general, studies have identified a significant correlation between MRI measures and CI [7, 39, 40] and, more specifically, studies suggest a robust correlation between cognitive deficits and irreversible tissue loss in the brain, usually measured in terms of global and regional atrophy [41]. In our study, fingolimod showed better results than IFN β-1b on all MRI parameters, brain atrophy and clinical parameters (relapse rate and EDSS score). This is supported by previous studies in which fingolimod was associated with early and consistent reduction in brain volume loss, compared with both placebo and intramuscular IFN β-1a [42]. On the other hand, both fingolimod and IFN β-1b improved all cognitive domains affected by MS, as evaluated through the various tests of the BRB, with some differences in the improvement pattern. Apart from the confounding effects of the difference in baseline severity and drop-out patterns across the two groups, which hold true both for the effects on cognitive scales and on MRI, this difference in effects on MRI versus BRB may also be due to other factors, including better sensitivity of MRI (the most sensitive tool to capture early tissue loss quantitatively and independent of any practice effects [43]) compared with cognitive scales for the detection of changes in endpoints supposed to be correlated (such as CI and atrophy), or to different sensitivity across the individual scales to changes in similar or different cognitive domains. Finally, the limited duration of the trial (18 months) may have impacted on the possibility of observing the complete expression of differential effects between the treatment groups on cognitive impairment scales reflecting the between-groups differences on MRI. Trials of longer duration may therefore be required to assess this aspect.

This absence of a firm correlation (also due to the high number of confounding effects and biases in assessing CI) also shows that effects of interventions (pharmacologic or non-pharmacologic) on functional cognitive capacity should be shown directly and cannot be deduced from effects on brain volumetric measures.

With regard to the safety results of the study, although this was a pilot study of limited size and, therefore, not really comparable to larger studies reported in the literature for both drugs, the GOLDEN study supported the established safety and tolerability profile of both drugs. The only SAE suspected of being related to the study treatment was a self-limiting, second-degree atrioventricular block after the first-dose of fingolimod, which is consistent with the known first-dose cardiovascular effects of the drug. Apart from this, first-dose monitoring was relatively eventless, although some patients required extended monitoring for precautionary measures due to lowered heart rate. AEs suspected of being related to treatment were in line with the tolerability and safety/tolerability profile of the two drugs as documented in the respective Summary of Product Characteristics or reported in the literature. No significant differences between fingolimod and IFN β-1b groups with regards to safety parameters were evident except for the higher number of adverse events in the fingolimod group, and the greater proportion of patients experiencing an MS relapse in the IFN β-1b group.

In conclusion, the results of the GOLDEN study suggest that both fingolimod and IFN β-1b treatments were associated with improvements in all cognitive parameters, with some differences in the improvement patterns. Despite a disadvantage in terms of baseline characteristics and drop-out patterns, fingolimod treatment demonstrated significantly better effects than IFN β-1b on MRI parameters and relapse rate.

Also, although limited in size, the GOLDEN study confirms the favourable benefit–risk profile of fingolimod reported in previous studies.

Management of cognitive decline in MS, which substantially alters patients’ quality of life and is the leading cause of occupational disability in patients with MS [44], is an area that still needs further research. Among others, additional studies are warranted to better understand the effects of DMTs on cognitive function and its correlation with underlying disease mechanisms.
